# One-step CRISPR-Cas9-mediated knockout of native TCRαβ genes in human T cells using RNA electroporation

**DOI:** 10.1016/j.xpro.2023.102112

**Published:** 2023-02-10

**Authors:** Donovan Flumens, Diana Campillo-Davo, Ibo Janssens, Gils Roex, Jorrit De Waele, Sébastien Anguille, Eva Lion

**Affiliations:** 1 Laboratory of Experimental Hematology, Vaccine & Infectious Disease Institute (VAXINFECTIO), Faculty of Medicine and Health Sciences, University of Antwerp, 2610 Wilrijk, Belgium; 2 Center for Oncological Research (CORE), Integrated Personalized and Precision Oncology Network (IPPON), 2610 Wilrijk, Belgium; 3 Division of Hematology, Antwerp University Hospital, Drie Eikenstraat 655, 2650 Edegem, Belgium; 4 Center for Cell Therapy & Regenerative Medicine, Antwerp University Hospital, 2650 Edegem, Belgium

**Keywords:** Cell culture, Cell isolation, Flow Cytometry/Mass Cytometry, Immunology, CRISPR, Tissue Engineering

## Abstract

To avoid mispairing between native and introduced T cell receptors (TCRs) and to prevent graft-versus-host disease in allogeneic T cell therapies, TCRα and TCRβ chains of native TCRs are knocked out via CRISPR-Cas9. We demonstrate the isolation and activation of CD8^+^ T cells followed by electroporation of T cells with *in vitro* transcribed eSpCas9(1.1)-P2A-EGFP mRNA and single-guide RNAs targeting the TCRα and TCRβ constant regions. We then describe a flow cytometric analysis to determine TCR knockout efficiency.

## Highlights

• Non-viral RNA-based CRISPR protocol for T cell receptor (TCR) α and β knockout
• Highly efficient and scalable native TCR disruption in primary human activated T cells
• One-step single electroporation strategy, combinable with transgenic TCR engineering

## Abstract (editor highlights)

Publisher’s note: Undertaking any experimental protocol requires adherence to local institutional guidelines for laboratory safety and ethics.

## Graphical abstract

**Figure:**
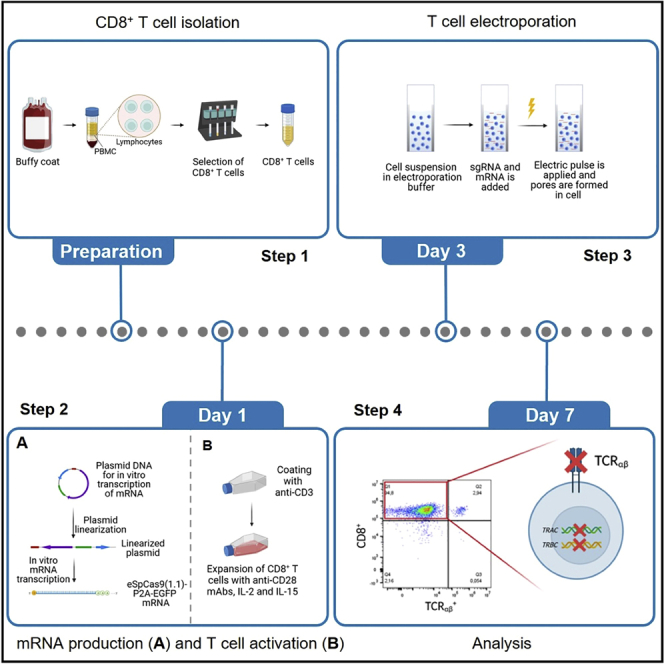

## Before you begin

The CRISPR-Cas9 system has revolutionized the field of molecular biology as a versatile genome-editing tool with a broad range of applications. Guided by a short RNA molecule, Cas9 is targeted to a genomic locus and creates a double-strand break. Upon cleavage by Cas9, the targeted locus is repaired by the dominant non-homologous end joining (NHEJ) pathway. This error-prone repair mechanism re-ligates the double-strand brakes, introducing small insertions or deletions (indels) at the breakpoint. Indels within a coding exon of the gene can lead to frameshift mutations and premature stop codons, resulting in a knockout of the gene.^1^ Although the CRISPR-Cas9 system is more accurate and efficient than other genome editing methods, integrating and stable delivery systems to introduce Cas9, for example those based on viral vectors, raise concerns about persistent Cas9 expression that could lead to off-target editing. Thus, non-viral methods that involve transient expression of Cas9, such as those using Cas9 ribonucleoproteins (RNPs) or Cas9 mRNA, benefit from a better safety profile. Compared to Cas9 RNPs, in-house production of Cas9 mRNA using plasmid vectors usually requires less resources and more accessible infrastructure in the context of clinical translation, making mRNA electroporation a desirable method for non-viral T-cell engineering.^2^ This protocol describes an optimized single electroporation fully RNA-based CRISPR-Cas9 strategy to eliminate the native TCR genes. Purified CD8^+^ T cells are activated with anti-CD3 and anti-CD28 antibodies for three days. *In vitro* transcribed eSpCas9(1.1)-P2A-EGFP mRNA is co-electroporated with single guide RNAs (sgRNAs) specific for human TCR α-chain constant (*TRAC*) and TCR β-chain constant (*TRBC*) in activated T cells to create double-strand breaks in the native TCR loci. The resulting TCR knockout can prevent TCR mispairing between native and introduced TCRs as well as GvHD in allogeneic T-cell therapies. In addition to CD8^+^ T cells, this protocol can also be applied to CD4^+^ T cells.

### Institutional permissions

All procedures involving human blood samples should be performed in accordance with relevant institutional and governmental ethics regulations. Informed consent should be obtained from all subjects for the experimental use of human blood. In this case, experimental work was compliant with the local Ethics Committee of the Antwerp University Hospital-University of Antwerp (Antwerp, Belgium) under project ID 0511. Selection of blood donors and collection of blood was performed according to Belgian law and Belgian Red Cross policy. This includes the signing of an informed consent in which the donor agrees that his/her blood can be used for purposes other than blood transfusion, including experimental research.

### Peripheral blood mononuclear cells (PBMC) isolation from buffy coats

**Timing: 3 h**

This step describes how to isolate PBMC from (healthy) donor buffy coats. Buffy coats primarily contain leukocytes and platelets and are derived from whole blood donations of anonymous volunteers. In this protocol, donor buffy coats were provided by the Blood Service of the Donor Center Mechelen (Red Cross-Flanders, Mechelen, Belgium). Density gradient centrifugation is used to separate the mononuclear leukocytes from the rest fraction of red blood cells (RBC), polymorphonuclear leukocytes (granulocytes) and platelets. The protocol below applies Ficoll-Paque PLUS as density gradient medium and has an estimated yield of 6.5 × 10^8^ PBMCs per 40 mL buffy coat. Alternatively, other density gradient media can be used to isolate PBMC, such as Lymphoprep (Stemcell Technologies).

1. Prepare phosphate-buffered saline (PBS)/Ethylenediaminetetraacetic acid (EDTA) buffer (see materials and equipment) and pre-heat to 37°C.
2. Wipe the blood tube of the buffy coat bag with 70% ethanol solution and cut the tube to release the blood.

**CRITICAL:** For this point on, sterile working is important to avoid contamination of the cells.

3. Divide the buffy coat over sterile 50 mL conical centrifuge tubes (∼10 mL blood per tube).
4. Dilute the blood 1:3 by adding pre-heated PBS/EDTA buffer to each tube. For 10 mL of blood, add to a final volume of 30 mL. Add the PBS/EDTA buffer fast enough to ensure good mixing.

***Note:*** When using whole blood as starting material instead of buffy coat preparation, 1:2 dilution is recommended.

5. Carefully layer 12 mL of Ficoll-Paque PLUS under the diluted buffy coat.

**CRITICAL:** Mixing of Ficoll-Paque PLUS and diluted blood must be avoided to obtain a high purity of PBMC.

***Note:*** Less Ficoll-Paque PLUS can be used but it may hinder the separation between the PBMC layer and the RBC fraction.

6. Centrifuge the tubes at 740 × *g* for 30 min (min) at 19°C–22°C in a swinging bucket rotor.

**CRITICAL:** For Ficoll-layered blood samples, lower the acceleration speed of the centrifuge and inactivate the rotor brakes to effectively prevent mixing of the different phases obtained after centrifugation.

***Note:*** After density gradient centrifugation each tube contains four layers. The top layer consists of plasma and platelets, followed by an opaque interphase with the PBMC, next the transparent Ficoll layer, and the RBC fraction at the bottom of the tube.

7. Collect the PBMC layer.
  a. Remove two thirds of the upper plasma layer, leaving a small volume of plasma on the PBMC interphase.
  b. Carefully loosen up the PBMC sticking to the side of the tube with the tip of your 10 mL pipette.
  c. Aspirate the PBMC layer and transfer to a clean, sterile 50 mL Falcon tube. Discard the remaining Ficoll and RBC layer.
  d. Repeat these steps for each tube.
8. Add PBS/EDTA to a final volume of 40 mL and centrifuge the tubes at 480 × *g* for 5 min at 19°C–22°C. Centrifugation can be performed with maximal acceleration and the rotor brakes on.
9. Discard the supernatant. Pool the cells to 2 × 50 mL tubes by resuspending the pellets in 10 mL PBS/EDTA.
10. Repeat washing step 8.
11. Discard the supernatant and pool the cells to 1 × 50 mL tube in a final volume of 50 mL PBS/EDTA.
12. After checking cell concentration with an automatic cell counter, transfer 100,000 PBMC to a polystyrene FACS tube to determine viability.
  a. Add 100 μL FACS buffer to the cells. Stain cells with 0.5 μL propidium iodide (PI; 1 mg/mL) and incubate for 1 min at 19°C–22°C.
  b. Measure cell viability on a flow cytometer with a filter set compatible with PI. A viability of 95%–99% is expected after PBMC isolation.

***Note:*** RBC contamination is a common issue with PBMC isolation and can cause potential errors in calculation of the cell concentration and downstream primary CD8^+^ T-cell isolation. RBC lysis buffer can be used to eliminate the RBC from the PBMC.

**Pause point:** Isolated PBMC can be kept for 16 h on a roller shaker in serum-free AIM-V medium at 1–1.6 × 10^7^ cells/mL or can be cryopreserved at 0.5–1 × 10^8^ cells/mL in 90% FBS + 10% dimethyl sulfoxide (DMSO) stored in a −80°C freezer for short-term storage or below −150°C for long-term storage.

### Positive magnetic-activated cell sorting (MACS) of primary resting CD8^+^ T cells

**Timing: 3 h**

The following section describes the immunomagnetic selection of primary resting CD8^+^ T cells from isolated human PBMC with human CD8 magnetic microbeads, according to the manufacturer instructions (Miltenyi). Generally, cytotoxic CD8^+^ T cells comprise 5%–25% of PBMC. With immunomagnetic selection, CD8^+^ T cells can be isolated from the unwanted cells with an expected purity of more than 95%.

***Note:*** Alternatively to positive selection, negative selection kits (e.g., Miltenyi or STEMCell) can be used to isolate CD8^+^ T cells.

13. Determine the percentage CD3^+^CD8^+^ T cells present in the PBMC.
  a. Add 100,000 PBMC to a polystyrene FACS tube.
  b. Wash the sample by adding 2 mL of FACS buffer.
  c. Centrifuge cells at 480 × *g* for 5 min at 19°C–22°C. Discard the supernatant.
  d. Resuspend the cell pellet in 100 μL of FACS buffer and stain the cell pellet with 5 μL anti-CD8 FITC and 5 μL anti-CD3 PerCP antibodies.
  e. Incubate 15 min at 19°C–22°C in the dark.
  f. Wash the sample by adding 2 mL of FACS buffer.
  g. Centrifuge cells at 480 × *g* for 5 min at 19°C–22°C. Discard the supernatant.
  h. Resuspend cell pellet in 100 μL FACS buffer.
  i. Acquire the stained cells on a flow cytometer and determine the percentage of CD3^+^CD8^+^ T cells (Figure 1A).

**Figure 1:**
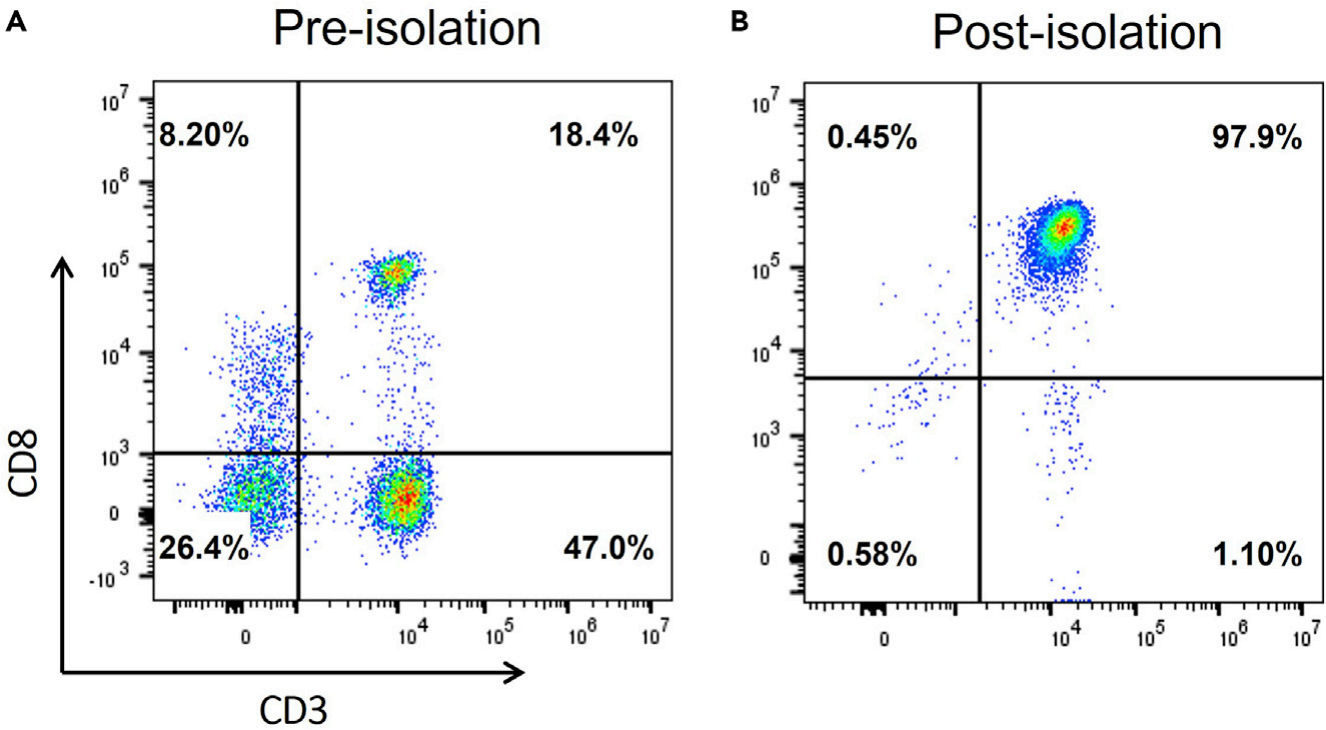
Representative flow cytometric plots of the percentage of CD8^+^ cells in freshly isolated PBMC (A) and after immunomagnetic enrichment (B) Gates were set on viable cells gating on the forward scatter (FSC) and side scatter (SSC) pseudocolor plots. CD8^+^ CD3^+^ double positive cells are identified as CD8^+^ T cells. A purity of more than 95% can be expected after immunomagnetic isolation.
14. Calculate the amount of PBMC to be used for the desired number of CD8^+^ T cells to be isolated. Transfer the PBMC to a clean, sterile 50 mL Falcon tube.
15. Centrifuge cell suspension at 300 × *g* for 10 min at 19°C–22°C. Remove supernatant completely.
16. Follow instructions on the manufacturer’s protocol.

**CRITICAL:** From this step on, work, fast, keep cells cold, and use pre-cooled solutions. This will prevent capping of antibodies on the cell surface and non-specific cell labeling.

**CRITICAL:** When flushing the cells of the LS column, extra caution is required when removing the LS column from the magnetic MACS separator and placing it into a 15 mL tube to avoid contamination due to manual handling.

17. Determine the yield and purity of the isolated CD8^+^ T cells.
  a. Take 100 μL isolated CD8^+^ T cells and divide over two polystyrene FACS tubes (50,000 cells/tube). Use one tube to determine the cell concentration on an automatic cell counter and to check cell viability with PI (see step 12). A viability ranging from 90%–99% can be expected. Use the other tube to check the purity of the isolation (% of CD3^+^CD8^+^ T cells).
  b. Wash the samples by adding 2 mL of FACS buffer.
  c. Centrifuge cells at 480 × *g* for 5 min at 19°C–22°C.
  d. Discard the supernatant.
  e. Stain the cell pellet with 5 μL anti-CD8 FITC and 5 μL anti-CD3 PerCP antibodies.
  f. Incubate 15 min at 19°C–22°C in the dark.
  g. Wash the sample by adding 2 mL of FACS buffer.
  h. Centrifuge cells at 480 × *g* for 5 min at 19°C–22°C.
  i. Discard the supernatant.
  j. Resuspend cell pellet in 100 μL FACS buffer.
  k. Acquire the stained cells on a flow cytometer and determine the percentage of CD3^+^CD8^+^ T cells (Figure 1B) (problem 1).

**Pause point:** Isolated CD8^+^ T cells can be cryopreserved at 10–50 × 10^6^ in 1 mL of 90% FBS + 10% DMSO stored in a −80°C freezer for short-term or below −150°C for long-term storage. It is not recommended to cryopreserve CD8^+^ T cells when starting from cryopreserved PBMC.

## Key resources table

**Table:** 

REAGENT or RESOURCE	SOURCE	IDENTIFIER
**Antibodies**		

Mouse anti-human CD3, PerCP, Clone SK7 (5 μL/10^5^ cells)	BD Biosciences	Cat# 345766 RRID: AB_2783791
Mouse anti-human CD4, PE, Clone SK3 (5 μL/10^5^ cells)	BD Biosciences	Cat# 345769 RRID: AB_2728699
Mouse anti-human CD8, FITC, Clone SK1 (5 μL/10^5^ cells)	BD Biosciences	Cat# 345772 RRID: AB_2868800
Mouse anti-human TCRα/β, PE, Clone IP26 (5 μL/10^5^ cells)	BioLegend	Cat# 306708 RRID: AB_314646
NA/LE mouse anti-human CD3, unconjugated, (5 μg/mL)	BD Biosciences	Cat# 555329 RRID: AB_395736
NA/LE mouse anti-human CD28, unconjugated, (1 μL/mL)	BD Biosciences	Cat# 555725 RRID: AB_396068

**Biological samples**		

Unpurified human buffy coat (25–50 mL) from healthy donors (both male and female human subjects, ages range from 18–70)	Flemish Red Cross	N/A

**Chemicals, peptides, and recombinant proteins**		

Dulbeco’s PBS without Ca^2+^ and Mg^2+^	Life Technologies	Cat# 14200067
Ethylenediaminetetraacetic acid (EDTA)-Na_2_ salt	Merck	Cat# 1084180250
Sodium azide (NaN_3_)	Merck	Cat# 769320
Ammonium chloride (NH_4_Cl)	Merck	Cat# 101145
Potassium bicarbonate (KHCO_3_)	Merck	Cat# 104854
Ficoll-Paque PLUS density gradient media	GE Healthcare	Cat# 17-1440-03
Bovine serum albumin (BSA)	Sigma-Aldrich	Cat# A1662-1L
IMDM medium	Life Technologies	Cat# 21980032
Human serum albumin (hAB)	Sanbio	Cat# A25761
Human recombinant interleukin 2 protein (rhIL-2)	ImmunoTools	Cat# 11340025
Human recombinant interleukin 15 protein (rhIL-15)	ImmunoTools	Cat# 11340155
Opti-MEM I reduced serum medium	Life Technologies	Cat# 11058021
Fetal bovine serum (FBS)	Life Technologies	Cat# 10270106
Dimethyl sulfoxide (DMSO)	Sigma-Aldrich	Cat# D2650-100mL
DNA Away Surface Decontaminant solution	Life Technologies	Cat# 7010PK
LguI (SapI) restriction enzyme	Thermo Fisher Scientific	Cat# ER1932
Tango buffer	Thermo Fisher Scientific	Cat# BY5
3 M Sodium acetate (pH 5.2)	Thermo Fisher Scientific	Cat# R1181
Absolute ethanol (for molecular biology)	Sigma-Aldrich	Cat# 1.08543.0250
Nuclease-free UltraPure water	Invitrogen	Cat# 10977035
TrackI 1 kb Plus DNA Ladder	Thermo Fisher Scientific	Cat# 10488085
ssRNA ladder	New England Biolabs	Cat# N0362S
Propidium iodide (1 mg/mL)	Life Technologies	Cat# P3566
BD FACSFlow	BD Biosciences	Cat# 342003

**Critical commercial assays**		

CD8 MicroBeads	Miltenyi Biotec	Cat# 130-045-201
mMESSAGE mMACHINE T7 Transcription Kit (2× NTP/CAP, 10× Reaction buffer, T7 Enzyme mix, TURBO DNase, and LiCl Precipitation Solution are included in the kit)	Thermo Fisher Scientific	Cat# AM1344

**Deposited data**		

Plasmid map	This paper	

**Oligonucleotides**		

CRISPRevolution sgRNA EZ Kit (100 nmol) - TRAC (exon 1) - Modified	Synthego	(5′-AGAGUCUCUCAGCUGGUACA -3′)
CRISPRevolution sgRNA EZ Kit (100 nmol) - TRBC (exon 1) - Modified	Synthego	(5′-GGAGAAUGACGAGUGGACCC -3′)

**Recombinant DNA**		

pST1 eSpCas9(1.1)-P2A-EGFP	Gifted by Prof./This paper	pST1 eSpCas9(1.1)-P2A-EGFP

**Software and algorithms**		

FlowJo v.10.8.0	FlowJo LLC	https://www.flowjo.com/
GraphPad Prism 9.3.1	GraphPad Software, LLC	https://www.graphpad.com/scientific-software/prism/
CytExpert 2.3.0.84	Beckman Coulter	https://www.beckman.pt/flow-cytometry/research-flow-cytometers/cytoflex/software

**Other**		

CELLSTAR® T75 Flask culture flask	Greiner Bio-One	Cat# 658175
CELLSTAR® 15 mL Falcon tubes	Greiner Bio-One	Cat# 188271
CELLSTAR® 50 mL Falcon tubes	Greiner Bio-One	Cat# 227261
LS column	Miltenyi Biotec	Cat# 130-042-401
QuadroMACS Separator	Miltenyi Biotec	Cat# 130-091-051
MACS MultiStand (magnetic stand)	Miltenyi Biotec	Cat# 130-042-303
Polystyrene FACS tubes	MLS	Cat# A10065N
Electroporation cuvettes	Immunosource	Cat# EP-104
Gene Pulser Xcell Electroporation system	BioRad	N/A
ABX Micros ES60 Hematology Analyzer	Horiba	N/A
CytoFLEX Flow Cytometer	Beckman Coulter	N/A
NanoDrop One Microvolume UV-Vis Spectrophotometer	Thermo Fisher Scientific	ND-ONE-W

## Materials and equipment

**Table:** PBS (1×) buffer

Reagent	Final concentration	Amount
ddH_2_O	N/A	900 mL
PBS (10×)	1×	100 mL
**Total**	**N/A**	**1 L**

Filter sterilize, keep sterile, store at 4°C for up to 3 months.

**Table:** EDTA buffer

Reagent	Final concentration	Amount
ddH_2_O	N/A	1 L
EDTA-Na2 salt	100 mM	37.224 g
**Total**	**N/A**	**1 L**

Filter sterilize, keep sterile, store at 4°C for up to 12 months.

**CRITICAL:** EDTA causes skin and severe eye irritation. Always wear gloves, goggles, and lab coat while handling it.

**Table:** PBS/EDTA buffer

Reagent	Final concentration	Amount
PBS (1×)	N/A	990 mL
EDTA buffer (100 mM)	1 mM	10 mL
**Total**	**N/A**	**1 L**

Filter sterilize, keep sterile, store at 4°C for up to 3 months.

**Table:** MACS buffer

Reagent	Final concentration	Amount
PBS (1×)	N/A	963.25 mL
EDTA buffer (100 mM)	2 mM	20 mL
Bovine serum albumin (30%)	10 nM	16.75 mL
**Total**	**N/A**	**1 L**

Filter sterilize, keep sterile, store at 4°C for up to 3 months.

**Table:** Sodium azide stock solution (1% w/v)

Reagent	Final concentration	Amount
ddH_2_O	N/A	1 L
Sodium azide (NaN_3_)	1%	10 g
**Total**	**N/A**	**1 L**

Filter sterilize, keep sterile, store at 19°C–22°C for up to 12 months.

**CRITICAL:** NaN_3_ is deadly by oral ingestion, dermal contact and inhalation. Always wear gloves, goggles, and lab coat while handling it and work under a fume hood. It is very toxic to aquatic life and with long lasting effects. Avoid release to the environment.

**Table:** FACS buffer

Reagent	Final concentration	Amount
BD FACSFlow	N/A	946.7 mL
Bovine serum albumin (30%)	0.1%	3.3 mL
Sodium azide (NaN_3_; 1%)	0.05%	50 mL
**Total**	**N/A**	**1 L**

Store at 4°C for up to 6 months.

**Table:** Lysis buffer (10×) stock solution

Reagent	Final concentration	Amount
ddH_2_O	N/A	1 L
Ammonium chloride (NH_4_Cl)	1,55 M	82.9 g
Potassium bicarbonate (KHCO_3_)	0,1 M	10 g
EDTA-Na_2_ salt	1 mM	370 mg
**Total**	**N/A**	**1 L**

Filter sterilize, keep sterile, store at 4°C for up to 12 months.

**CRITICAL:** NH_4_Cl is toxic by oral ingestion.

**Table:** Lysis buffer (1×) working solution

Reagent	Final concentration	Amount
ddH_2_O	N/A	45 mL
Lysis buffer (10×) stock solution	1×	5 mL
**Total**	**N/A**	**50 mL**

Filter sterilize, keep sterile, store at 4°C for up to 6 months.

**Table:** 70% ethanol solution

Reagent	Final concentration	Amount
Nuclease-free Utra-Pure water	N/A	3 mL
Ethanol absolute	70%	7 mL
**Total**	**N/A**	**10 mL**

Store at 19°C–22°C for up to 3 months.

**CRITICAL:** Ethanol absolute is highly flammable.

**Table:** CTL medium

Reagent	Final concentration	Amount
IMDM cell culture medium	95%	47.5 mL
hAB	5%	2.5 mL
**Total**	**N/A**	**50 mL**

Keep sterile, store at 4°C for up to 3 months.

**Table:** Complete CTL medium

Reagent	Final concentration	Amount
IMDM cell culture medium	95%	47.5 mL
hAB	5%	2.5 mL
rhIL-2 (10 UI/μL)	50 UI/mL	0.25 mL
rhIL-15 (1 ng/μL)	10 ng/mL	0.5 mL
**Total**	**N/A**	**50 mL**

Keep sterile, store at 4°C for up to 7 days; IL-2 degrades easily in the medium.

**Table:** Electroporation (EP) recovery medium

Reagent	Final concentration	Amount
IMDM cell culture medium	90%	45 mL
hAB	10%	5 mL
**Total**	**N/A**	**50 mL**

Keep sterile, store at 4°C for up to 3 months.

**Table:** Aliquots of *TRAC* sgRNA

Reagent	Final concentration	Amount
Nuclease-free UltraPure water	N/A	3.2454 mL
*TRAC* sgRNA (32,454 g/mol)	1 μg/μL	100 nmol
**Total**	**N/A**	**3.2454 mL**

Make aliquots of 40 μL in nuclease-free tubes. Keep sterile, store at −20°C for up to 12 months.

**Table:** Aliquots of *TRBC* sgRNA

Reagent	Final concentration	Amount
Nuclease-free UltraPure water	N/A	3.2495 mL
*TRBC* sgRNA (32,495 g/mol)	1 μg/μL	100 nmol
**Total**	**N/A**	**3.2495 mL**

Make aliquots of 40 μL in nuclease-free tubes. Keep sterile, store at −20°C for up to 12 months.

## Step-by-step method details

### Design of pST1 eSpCas9(1.1)-P2A-EGFP plasmid

**Timing: 1 day**

**Figure 2:**
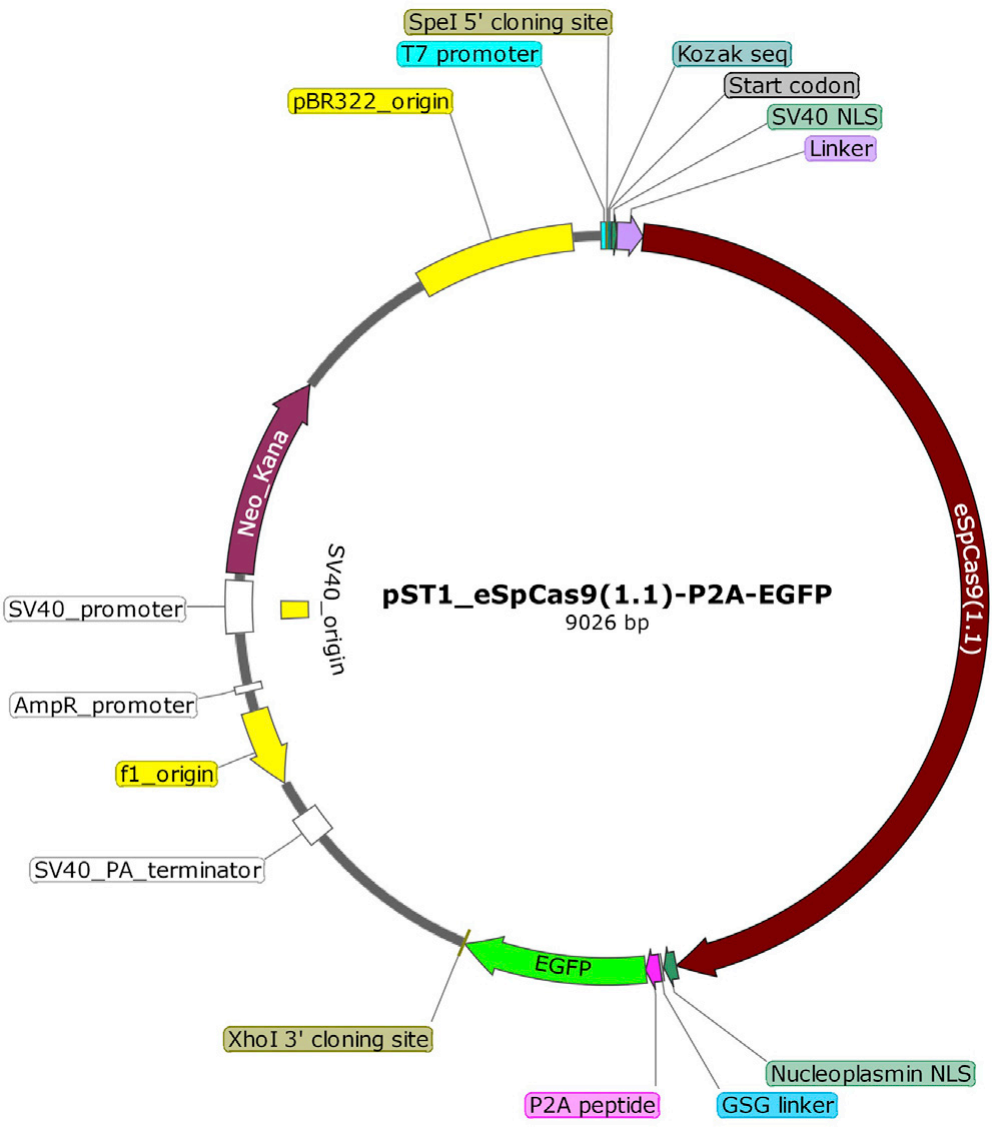
Schematic representation of the pST1 eSpCas9(1.1)-P2A-EGFP plasmid vector A pST1 plasmid vector containing rationally designed eSpCas9(1.1)^3^ linked to an enhanced green fluorescent protein (EGFP) reporter was designed for *in vitro* production of Cas9 mRNA. The plasmid vector representation was created with SnapGene.

In order to delete native *TRAC* and *TRBC* genes in CD8^+^ T cells with a high degree of efficiency, a rationally engineered version of *Streptococcus pyogenes* Cas9 (SpCas9) with enhanced specificity (eSpCas9(1.1)) is used.^3^ The eSpCas9(1.1) sequence is inserted into a pST1 plasmid vector back-bone.^4^^,^^5^ This vector has been optimized for the *in vitro* transcription (IVT) of mRNA to rapidly and efficiently produce high amounts of synthetic mRNA. The open reading frame of the eSpCas9(1.1) is preceded by a T7 promoter, a 5′ cloning site, a Kozak sequence at the translational start site, and one copy of the monopartite nuclear localization signal of the simian virus 40 (SV40) T-antigen (PKKKRKV) followed by a 32 amino-acid linker.^6^ The eSpCas9(1.1) is followed by a copy of the bipartite NLS from nucleoplasmin, a GSG linker, a 2A peptide from a porcine teschovirus-1 (P2A) and an enhanced green fluorescence protein (EGFP) reporter gene and the 3′ cloning site. The P2A sequence allows co-expression of both eSpCas9(1.1) and EGFP by self-cleavage of the P2A peptide during translation.^7^ An EGFP reporter gene is added to the construct to determine the transfection efficiency of the mRNA construct after electroporation and can be easily omitted or replaced by a preferred reporter gene. The SV40 NLS-Linker-eSpCas9(1.1)-Nucleoplasmin NLS-GSG-P2A-EGFP (in short, eSpCas9(1.1)-P2A-EGFP) insert has a total length of 5070 nucleotides and was codon optimized for *Homo sapiens.* The 3′ untranslated region (UTR) present in the pST1 backbone is comprised of two copies of the 3′ UTR from the human alpha globin gene and a 120-nucleotide poly(A) tail, which increase the stability and translational efficiency of the produced mRNA,^4^ and a restriction site for a Type IIS restriction enzyme (Figure 2).

1. Design the eSpCas9(1.1)-P2A-EGFP DNA construct plasmid *in silico* by combining the segments described above in a plasmid vector of preference.
2. Construct synthesis, cloning and plasmid preparation can be performed in-house or outsourced to commercial providers such as Gene-Art.

***Optional:*** Any plasmid vector suitable for IVT of mRNA can be used as an alternative for the pST1 plasmid vector.

***Optional:*** The eSpCas9(1.1)-P2A-EGFP plasmid DNA can be amplified by transforming the plasmid into supercompetent *E. coli*. The pST1 vector contains both a bacterial origin of replication and a kanamycin resistance gene for use as a selectable marker in bacteria.

### Linearization of the pST1 eSpCas9(1.1)-P2A-EGFP plasmid

**Timing: 2 days**

For transcription of mRNA *in vitro*, the plasmid DNA template must be completely linearized with a restriction enzyme downstream of the 3′ UTR of the construct. Circular plasmid templates will generate extremely long, heterogeneous RNA transcripts because RNA polymerases are very processive. Completely linearized plasmid template of highest purity is critical for successful RNA synthesis, since the quality of the template DNA will affect transcription yield and integrity of the synthesized RNA. This step describes the linearization of a circular plasmid DNA using restriction enzymes and precipitation of the linear DNA prior to mRNA IVT.

3. Clean the DNA bench with DNA Away Surface Decontaminant solution to remove unwanted DNA and DNase.

**CRITICAL:** Avoid contact of this DNA Away Surface Decontaminant solution with your own plasmid.

**CRITICAL:** During the next steps it is important to work fast and use DNase-free filter pipette tips to avoid degradation of DNA.

4. Prepare the digestion reaction of plasmid DNA with restriction enzymes.
  a. Calculate the volumes of plasmid and water needed, according to the plasmid to be digested. For the pST1 plasmid vector, the LguI (SapI) restriction enzyme with a concentration of 5 U/μL is used. Approximately, 1U of enzyme cuts 1 μg of DNA in 1 h.
  b. Homogenize the plasmid DNA and the Tango buffer and short spin both tubes. Keep the DNA and the Tango buffer on ice.
  c. Add the reagents to a sterile 1.5-mL microcentrifuge tube in following order:

**Table:** 

Order	Reagent	Amount
1	Nuclease-free distilled water	Up to 500 μL
2	Plasmid DNA	50 μg
3	Tango buffer (10×)	50 μL
4	Restriction Enzyme	10 μL
  d. Mix the reagents by pipetting up and down and spin down the microcentrifuge tube. Do not vortex!
    ***Note:*** The volumes of the reagents can be downscaled, but in any case, the volume of the Tango buffer should be 1/10 of the final volume as it is a 10× solution.

**Figure 3:**
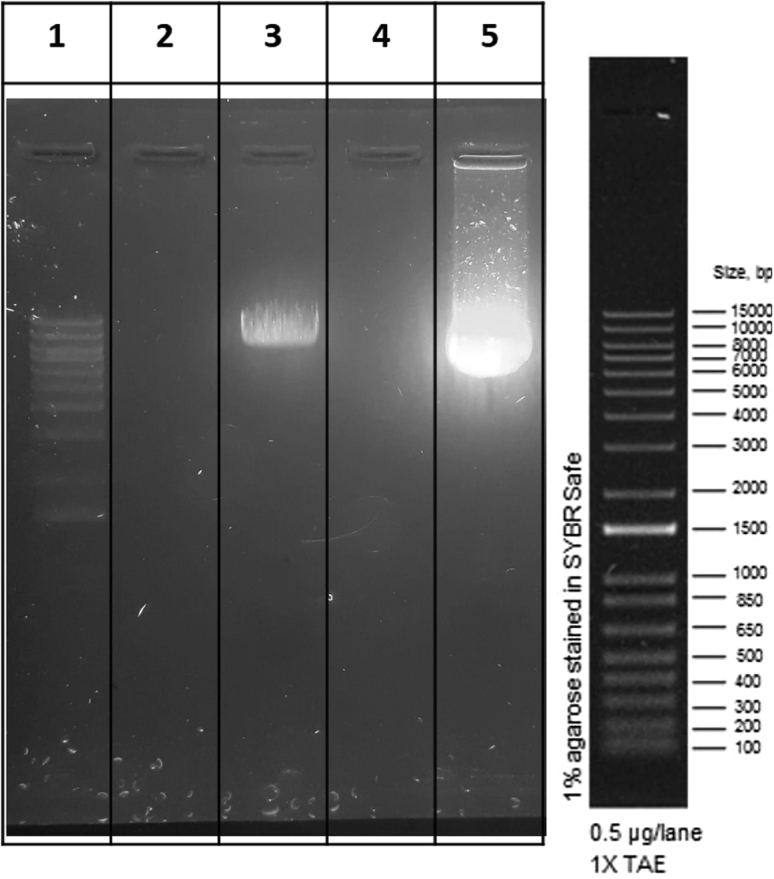
Representative 1% agarose gel showing successful linearization of pST1 eSpCas9(1.1)-P2A-EGFP plasmid (1) a TrackIt 1 kb Plus DNA Ladder (DNA ladder) is used for sizing of the linearized plasmid DNA. (3) A band representing linear pST1 eSpCas9(1.1)-P2A-EGFP. The linear plasmid DNA has a length of 9026 bases. (5) Non-linearized pST1 eSpCas9(1.1)-P2A-EGFP plasmid DNA. Column 2 and 4 are empty.

***Note:*** linearized plasmid DNA is precipitated with sodium acetate and ethanol solutions. Ethanol precipitation is a commonly used method to de-salt and concentrate DNA. Nucleic acids are negatively charged due to the presence of phosphate groups, making DNA readily soluble in water. Addition of sodium acetate and ethanol disrupts the hydrate shell and neutralizes the negative charge of DNA, leading to precipitation.

5. Incubate the microcentrifuge tube on a thermoblock for 2 h at 37°C.
6. Examine the linearized DNA template in a 1% agarose gel electrophoresis to confirm cleavage of the plasmid is complete (Figure 3).

7. Add 50 μL of 3 M sodium acetate (pH 5.2) to the microcentrifuge tube. This is 1/10 of the final volume used to linearize 50 μg of plasmid DNA.
8. Add 1 mL of absolute ethanol to the microcentrifuge tube. This is 2 volumes of the final volume used to linearize 50 μg of plasmid DNA.
9. Mix the solution gently by pipetting.
10. Incubate 16–24 h at −20°C.
11. Centrifuge the microcentrifuge tube at 16,100 × *g* for 15 min at 4°C.
12. Remove the supernatant carefully without disturbing the DNA pellet.
13. Add 1 mL of 70% ethanol solution to the DNA pellet. Do not resuspend the pellet.

***Note:*** Place the microcentrifuge tube in the same direction in the centrifuge for each centrifugation step in order to know where the DNA pellet is, since sometimes it is difficult to see.

14. Centrifuge the microcentrifuge tube at 16,100 × *g* for 15 min at 4°C.
15. Remove supernatant carefully without disturbing the DNA pellet.
16. Add 50 μL of nuclease-free distilled water to dissolve the DNA pellet.

**Caution:** Do not pipette up and down the DNA pellet. Instead, leave it for 30 min or longer at 4°C to dissolve.

17. Determine the concentration of linearized plasmid DNA with a NanoDrop (Thermo Fisher) and bring the linearized plasmid DNA to a concentration of 0.5 μg/μL. Approximately 90% of the starting material is recovered after linearization and precipitation.

**Pause point:** Linear DNA can be directly used for IVT of mRNA or stored at −20°C for at least 1 year.

### *In vitro* transcription of eSpCas9(1.1)-P2A-EGFP mRNA

**Timing: 7 h**

The eSpCas9(1.1)-P2A-EGFP mRNA is synthesized by IVT using the mMESSAGE mMACHINE T7 transcription kit. The protocol described below is adapted from the manufacturer’s recommendations (Thermo Fisher Scientific) and uses a T7 enzyme mix containing RNA polymerases to produce large amounts of capped RNA. According to the manufacturer, this kit produces an average yield of 20–30 μg mRNA per 1 μg of linearized plasmid template and the reaction setup showed here is compatible with mRNA of 300 bases to 5 kb in length.

18. Thaw the frozen agents of the mMESSAGE mMACHINE T7 transcription kit.

***Note:*** Keep the RNA Polymerase Enzyme Mix and 2× NTP/CAP on ice and keep the 10× Reaction buffer at 19°C–22°C while assembling the reaction.

**CRITICAL:** During the next steps it is important to work fast and use RNase-free filter pipette tips to avoid degradation of the RNAs.

19. Vortex the 10× Reaction buffer and 2× NTP/CAP.
20. Briefly spin all reagents to prevent contamination or loss of reagent.
21. Assemble the transcription reaction in a sterile 1.5 mL microcentrifuge tube at 19°C–22°C in the following order:

**Table:** 

Reagent	Volume (1×)
Nuclease-free distilled water	Up to 20 μL
2× NTP/CAP	10 μL
10× Reaction buffer	2 μL
Linearized plasmid DNA template	1 μg
T7 Enzyme Mix	2 μL

***Note:*** The table represents the volumes for 1 IVT reaction with 1 μg of linearized plasmid template. The reactions can be easily scaled up or down.

22. Gently mix by pipetting and centrifuge briefly.
23. Incubate the mix for 2 h at 37°C on a thermoblock.
24. Add 1 μL TURBO DNase per IVT reaction and mix well.
25. Incubate the solution for 15 min at 37°C on a thermoblock.
26. Stop the IVT reaction and precipitate the mRNA by adding 50 μL of LiCl Precipitation Solution per IVT reaction.
27. Mix thoroughly and incubate for more than 30 min at −20°C.

***Note:*** LiCl precipitation can remove unincorporated nucleotides and most proteins; however, it does not precipitate transfer RNA and it may not efficiently precipitate RNAs smaller than 300 nucleotides. The addition of LiCl will prevent freezing of the solution.

***Optional:*** LiCl precipitation can be performed overnight at −20°C.

28. Centrifuge the suspension at 16,100 × *g* for 15 min at 4°C.
29. Remove the supernatant carefully without disturbing the mRNA pellet.
30. Wash the mRNA pellet once with 1 mL of 70% ethanol solution and centrifuge at 16,100 × *g* for 15 min at 4°C. Do not resuspend the pellet.
31. Remove the supernatant carefully without disturbing the mRNA pellet.
32. Let the pellet dry with the cap open for 2–5 min at 19°C–22°C.
33. Add 10 μL of nuclease-free distilled water per IVT reaction and let the mRNA dissolve in the water at 4°C for ±30 min.
34. Determine mRNA concentration on Nanodrop. Keep the mRNA on ice during the measurement (problem 2).
35. Bring the concentration of the mRNA to 2 μg/μL.
36. Aliquot the mRNA in 20 μL per vial and freeze at −80°C. Frozen mRNA can be stored for 3 years.

**Figure 4:**
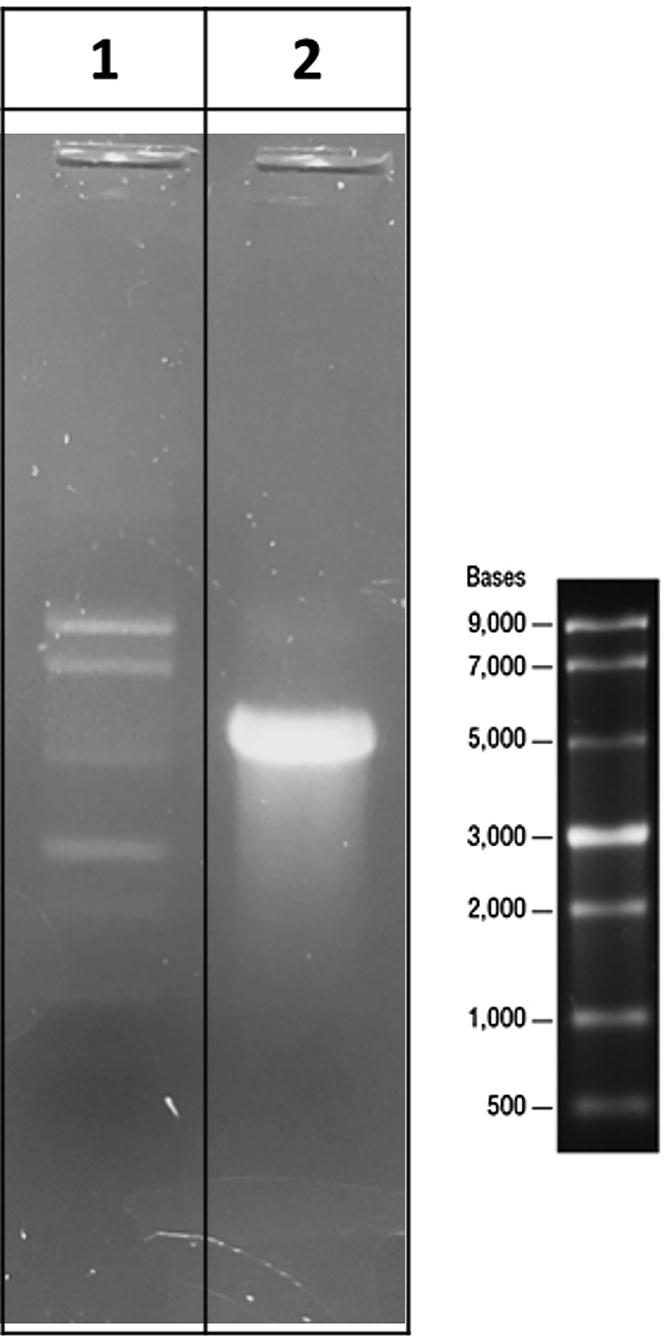
Representative 1% agarose gel for quality control of in IVT eSpCas9(1.1)-P2A-EGFP mRNA (1) A single stranded RNA ladder (ssRNA ladder) with a range of 500 bases to 9,000 bases is used for sizing the mRNA construct. (2) Resulting eSpCas9(1.1)-P2A-EGFP mRNA (mRNA Cas9 EGFP) has a length of 5500 nucleotides.

***Optional:*** As quality control, IVT mRNA can be examined in a non-denaturing 1% agarose gel electrophoresis to confirm the integrity and the length of the produced mRNA (Figure 4).

### Activation of primary CD8^+^ T cells

**Timing: 3 h**

The section below describes an *in vitro* activation protocol for primary CD8^+^ T cells via stimulation of the TCR-CD3 complex using plate-bound anti-CD3 and soluble anti-CD28 monoclonal antibodies and stimulatory cytokines. Short-term activation of T cells will enable greater RNA transfection efficiency compared to non-activated T cells and thus subsequent disruption of the native *TRAC* and *TRBC* sequences.

***Note:*** Both MACS-isolated fresh and cryopreserved human CD8^+^ T cells can be used. When using cryopreserved cells, thaw them in CTL medium and let the cells rest for at least 4 h before activation (problem 3).

37. Prepare a 5 μg/mL solution of no azide (NA)/low endotoxin (LE) anti-CD3 antibody in sterile PBS (1×) buffer. For a T75 cell culture flask, add 50 μL of NA/LE anti-CD3 antibody (1.0 mg/mL) to 10 mL of PBS (1×) buffer.
38. Add the solution to a T75 cell culture flask and incubate the flask horizontally in a humified incubator at 37°C and 5% CO_2_ for 2 h. Make sure that the entire surface is covered.

***Note:*** Volume of coating solution is calculated based on the surface area of the culture flask. Downscale or upscale the volume of 5 μg/mL NA/LE anti-CD3 antibody solution according to the surface area of the specific type of flask used for cell culture.

39. Aspirate the NA/LE anti-CD3 antibody solution and discard.
40. Carefully wash the anti-CD3 coated T75 cell culture flask with 10 mL of sterile PBS (1×) buffer. Avoid touching the coated surface of the flask.
41. Prepare 20 × 10^6^ viable primary CD8^+^ T cells.
42. Centrifuge the CD8^+^ T cells and discard the supernatant.
43. Add 20 mL complete CTL medium to bring cell concentration to 10^6^ cells/mL.
44. Transfer cells to the anti-CD3 coated T75 cell culture flask.
45. Add 20 μL NA/LE anti-CD28 (1.0 mg/mL) antibody to the culture flask.
46. Gently tilt the flask to homogenize the suspension.
47. Incubate flasks horizontally in a humidified incubator at 37°C and 5% CO_2_ for 3 days (problem 4).

***Note:*** Volumes are adjusted for 20 × 10^6^ primary CD8^+^ T cells at the beginning of the culture. Up to 30 × 10^6^ primary CD8^+^ T cells can be cultured in a T75 cell culture flask; in that case, adjust the volumes of NA/LE anti-CD28 antibody and cytokines accordingly.

### Generation of TKO8 cells with RNA electroporation

**Timing: 2 h**

mRNA electroporation is a powerful tool for transient genetic modification of cells.^2^ Combining the CRISPR gene editing tool with mRNA delivery provides a safer modification strategy compared to integrating delivery systems, for example those based on viral vectors.^8^ In this step, activated primary CD8^+^ T cells are electroporated with eSpCas9(1.1)-P2A-EGFP mRNA and two sgRNAs targeting exon 1 of *TRAC* and exon 1 of *TRBC* to disrupt the expression of native TCRs (referred to as TKO8 cells). After transfection into CD8^+^ T cells, translation of eSpCas9(1.1)-P2A-EGFP mRNA will result in transient eSpCas9(1.1) expression together with EGFP. Cas9 protein will then bind to the sgRNAs, which will be transported to the nucleus, and will be guided to the *TRAC* and *TRBC* sequences of interest by the sgRNAs; with this construct, EGFP operates as a reporter for transfection efficiency.

48. Prepare EP recovery medium and pre-heat 5 mL in a 15 mL tube in a 37°C water bath.
49. Carefully resuspend activated CD8^+^ T cells from the T75 flask by pipetting against the bottom of the flask.
50. Take 100 μL of activated cells to determine the cell concentration on an automatic cell counter and to check cell viability with PI (see step 12, before you begin). A viability ranging from 90%–99% can be expected.
51. Collect 10^7^ viable activated CD8^+^ T cells.

***Optional:*** This protocol can be upscaled up to 5 × 10^7^ cells in a maximum of 400 μL of electroporation buffer for a 4 mm electroporation cuvette.

52. Centrifuge CD8^+^ T cells at 480 × *g* for 5 min at 19°C–22°C. Remove supernatant completely.
53. Wash T cells by adding 10 mL cold serum-free phenol red-free Opti-MEM.
54. Centrifuge T cells at 480 × *g* for 5 min at 19°C–22°C. Remove supernatant completely.

**CRITICAL:** During the next steps it is important to work fast and use RNase-free filter pipette tips to avoid degradation of the RNAs.

55. Resuspend the cell pellet in 200 μL cold serum-free phenol red-free Opti-MEM.
56. Transfer the cell suspension to a sterile RNase-free microcentrifuge tube.
57. Thaw the eSpCas9(1.1)-P2A-EGFP mRNA and *TRAC* and *TRBC* sgRNAs. Keep them on ice or in a lab cooler at 0°C.

**CRITICAL:** mRNA constructs should be kept on ice at all times as RNA is susceptible to degradation when left at room temperature.

58. Add 10 μg of eSpCas9(1.1)-P2A-EGFP mRNA to the cell suspension (final mRNA concentration: 50 μg/mL).
59. Add 2.5 μg of *TRAC* sgRNA to the cell suspension (final sgRNA concentration: 12.5 μg/mL).
60. Add 2.5 μg of *TRBC* sgRNA to the cell suspension (final sgRNA concentration: 12.5 μg/mL).
61. Mix gently by pipetting up and down.
62. Transfer the cell suspension with RNAs to a 4-mm electroporation cuvette.

**CRITICAL:** The presence of bubbles in the electroporation cuvette or oil stains on the electrodes of the cuvettes due to handling without gloves can lead to arcing. This phenomenon is a complete or partial discharge of an electric current in a sample easily recognizable as an audible popping sound which negatively impacts cell viability.^2^

63. Set up the electroporation settings for transfection of T cells with RNA in the BioRad Gene Pulser Xcell electroporation system.
  a. Square wave electroporation protocol.
  b. Voltage: 500 V.
  c. Pulse length: 5 ms.
  d. Number of pulses: 1 pulse.
  e. Pulse intervals (sec): 0 s.
  f. Cuvette: 4 mm.
64. Gently shake the cuvette to evenly distribute the cell suspension and place the cuvette in the ShockPod cuvette chamber.
65. Press the pulse button to electroporate the cells.
66. After electroporation, transfer the CD8^+^ T cells immediately to the pre-heated EP recovery medium.
67. Incubate cells in a humified incubator for at least 2 h at 37°C and 5% CO_2_.
68. After 2 h, centrifuge CD8^+^ T cells at 480 × *g* for 5 min at 19°C–22°C. Remove supernatant completely.
69. Resuspend the cell pellet in 5 mL of complete CTL medium.
70. Take 100 μL of electroporated cells and transfer to a polystyrene FACS tube to determine the cell number and viability.
  a. Count cells with an automatic cell counter.
  b. Add FACS buffer up to 200 μL to the remaining cell suspension.
  c. Stain cells with 0.5 μL PI and incubate for 1 min at 19°C–22°C.
  d. Measure cell viability on a flow cytometer. Expected viability is ranging from 85%–95%.
71. Bring cell concentration to 0.3–1 × 10^6^ cells/mL with complete CTL medium in a T75 flask and keep in a humified incubator for 72 h at 37°C and 5% CO_2_, before validating TCR KO efficiency.

***Note:*** TKO8 cells can be further expanded immediately after electroporation, passaging the cells every 2–3 days with complete CTL medium, to obtain sufficient numbers for downstream applications. Due to the initial activation of primary CD8^+^ T cells with anti-CD3, anti-CD28 antibodies, IL-2 and IL-15, no second TCR stimulus is needed to further expand TKO8 cells. When expanding, keep TKO8 cell concentration at 0.3–1 × 10^6^ cells/mL. Transfer cells to larger cell culture flasks if needed. In addition, microscopic analysis of the cells can be performed to confirm the formation of T-cell clusters.

### Analysis of transfection efficiency

**Timing: 30 min**

**Figure 5:**
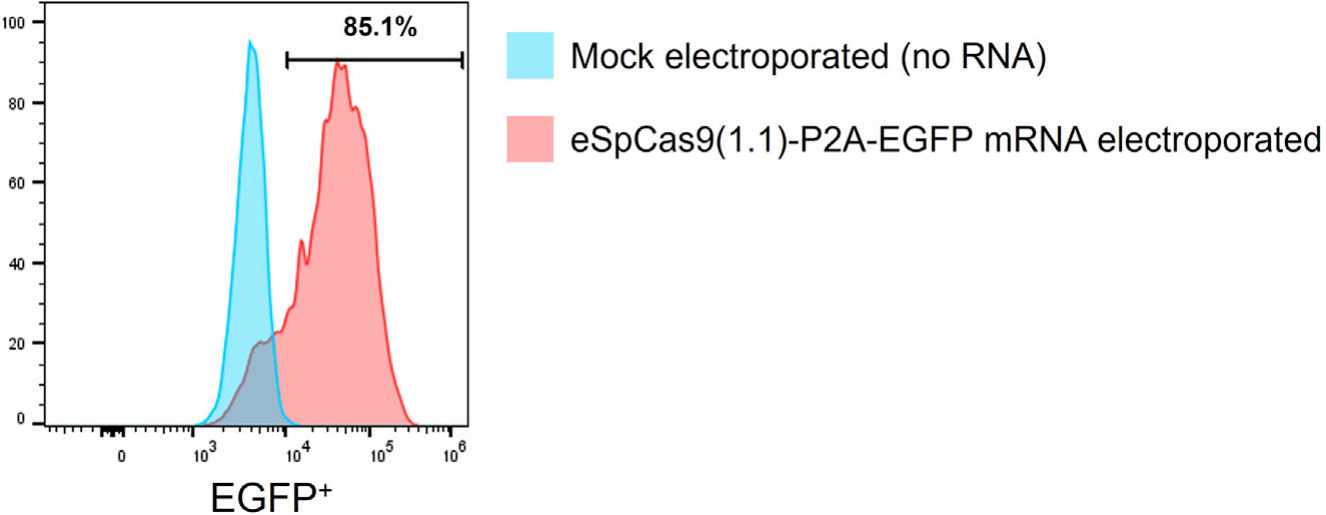
Flow cytometric analysis of EGFP expression as reporter for mRNA transfection efficiency Percentage of EGFP^+^ primary activated CD8^+^ T cells was evaluated 24 h after electroporation with eSpCas9(1.1)-P2A-EGFP mRNA and *TRAC* and *TRBC* sgRNAs. Expected transfection efficiency ranges from 80% to 90%. A mock electroporation without RNA was used as a negative control. EGFP^+^ cells were determined after gating on the viable cells.

Twenty-four hours after electroporation, transfection efficiency can be determined using flow cytometry by measuring the level of EGFP expression in the electroporated CD8^+^ T cells (Figure 5). Expression of EGFP peaks 24 h after electroporation and gradually decreases over time, with almost no expression 96 h after electroporation.

72. Take 100,000 of electroporated cells and transfer to a polystyrene FACS tube.
73. Add 2 mL FACS buffer and centrifuge the cells at 480 × *g* for 5 min at 19°C–22°C. Decant the supernatant after centrifugation.
74. Resuspend cell pellet in 100 μL FACS buffer.
75. Acquire the transfected cells on a flow cytometer and determine the percentage of EFGP^+^ T cells (Figure 5). Expected transfection efficiency (here demonstrated by EGFP^+^ cells) ranges from 80%–90%.

***Note:*** EGFP expression levels provide information about eSpCas9(1.1)-P2A-EGFP mRNA transfection efficiency in T cells after electroporation, as well as the correct translation of the mRNA, but it does not provide information regarding the efficiency of the editing.

### Validation of TCR knockout

**Timing: 30 min**

Stable TCR knockout efficiencies can be detected 72 h after electroporation. TCR knockout efficiency is validated by analyzing the surface expression of native TCR by flow cytometry using an anti-human TCRαβ monoclonal antibody. Additionally, expression levels of CD3ε are measured to confirm the TCR knockout, as CD3 can only be expressed in the presence of a TCR.

**Figure 6:**
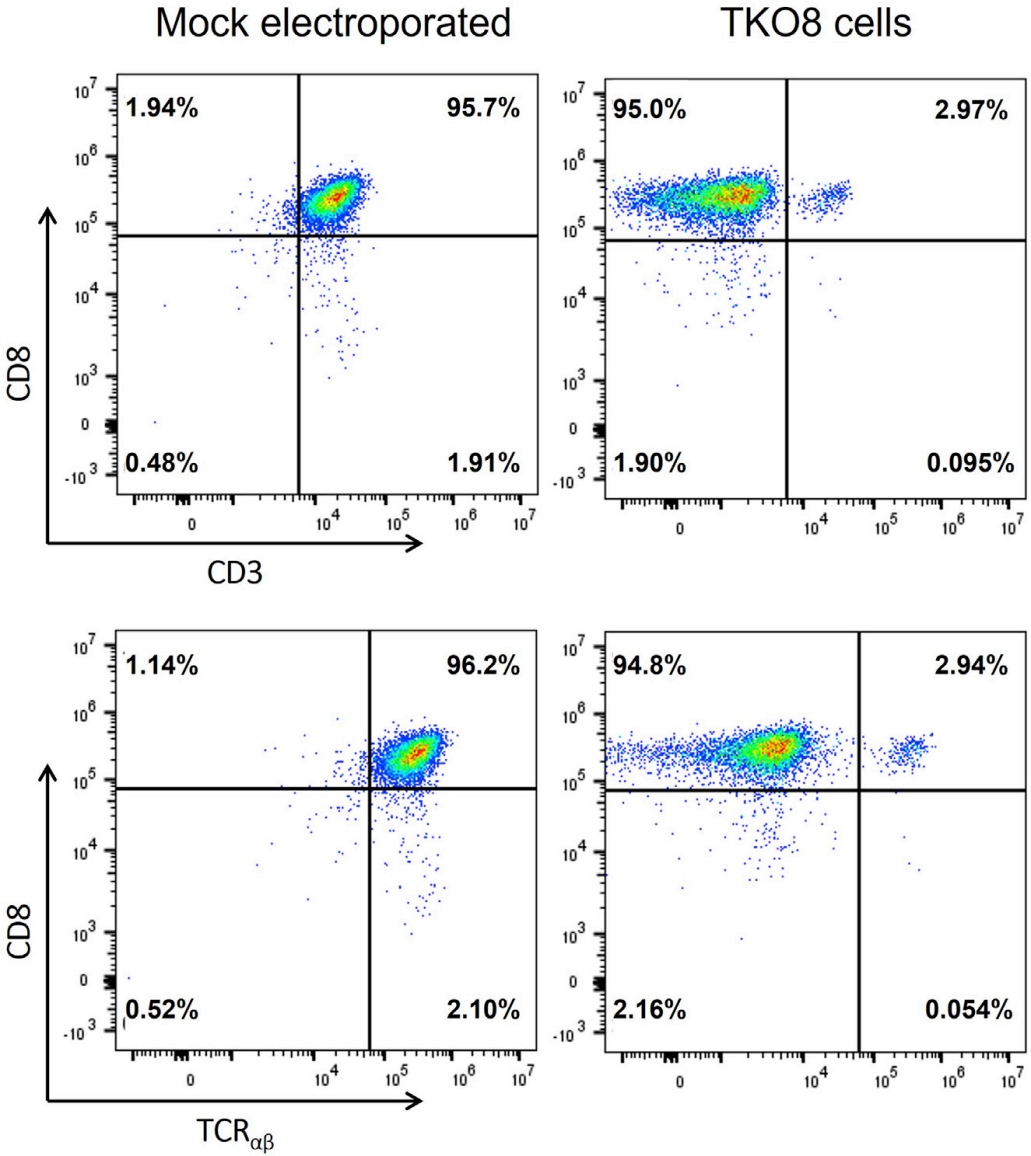
Representative flow cytometric plots of *TRAC* and *TRBC* KO in activated primary CD8^+^ T cells Native TCR KO CD8^+^ T cells (TKO8) cells were generated by electroporating primary 3-day activated CD8^+^ T cells with eSpCas9(1.1)-P2A-EGFP mRNA and *TRAC*- and *TRBC*-directed sgRNAs. A mock electroporation without Cas9 mRNA and sgRNAs was used as a negative control. TCR and CD3 expression is determined after gating on the viable cells.

***Note:*** Native TCR knockout efficiency could alternatively be assessed on a transcriptomic level using reverse transcription quantitative real-time PCR (RT-qPCR) or on a genomic level using DNA sequencing.

76. Take 100,000 electroporated cells and transfer to a polystyrene FACS tube.
77. Add 2 mL FACS buffer and centrifuge the cells at 480 × *g* for 5 min at 19°C–22°C. Decant the supernatant after centrifugation.
78. Resuspend cells in 100 μL FACS buffer and stain the cell pellet with 5 μL anti-CD8 FITC, 5 μL anti-TCRαβ PE and 5 μL anti-CD3 PerCP.
79. Incubate the cells for 15 min at 19°C–22°C in the dark.
80. Wash the sample by adding 2 mL of FACS buffer and centrifuge at 480 × *g* for 5 min at 19°C–22°C. Discard the supernatant.
81. Resuspend cell pellet in 100 μL FACS buffer.
82. Acquire the stained cells on a flow cytometer and determine the percentage of viable CD3^+^CD8^+^TCR^+^ T cells. A TCR knockout efficiency ranging from 90% to 99% can be expected (Figure 6) (problem 5).

## Expected outcomes

Viral methods to introduce Cas9 raise concerns about persistent Cas9 expression that could lead to off-target editing. Non-viral methods that involve transient expression of Cas9, such as those using Cas9 ribonucleoproteins or *Cas9* messenger RNA (mRNA), benefit from a better safety profile. Compared to Cas9 ribonucleoproteins, in-house production of *Cas9* mRNA using plasmid vectors usually requires less resources and more easily accessible infrastructure, making mRNA electroporation a desirable method for non-viral T-cell engineering. Here, IVT of eSpCas9(1.1)-P2A-EGFP mRNA with the mMESSAGE mMACHINE T7 transcription kit generally yields ±20 μg mRNA per 1 μg of linearized plasmid used for the reaction. This produced eSpCas9(1.1)-P2A-EGFP mRNA is then co-electroporated with sgRNAs specific for *TRAC* and *TRBC* in activated CD8^+^ T cells. With the described one-week single-electroporation RNA-based CRISPR-Cas9 protocol, TCR KO efficiency is expected to range from 90% to 99% 72 h after electroporation. Representative flow cytometric results after TCR KO are shown in Figure 6. 72 h after electroporation, native TCR expression is completely eliminated and TKO8 cells can be engineered to validate transgenic TCRs without chance of mispairing with the native TCR chains. In addition, this protocol could be applied in allogeneic T-cell therapy by non-viral and stable elimination of the native TCR to avoid GvHD. In summary, this RNA-based CRISPR/Cas9 strategy provides a robust and non-viral approach for rapid multiplex genome engineering of primary T cells.

## Limitations

This protocol provides an RNA-based method to efficiently disrupt native TCR expression in primary human CD8^+^ T cells. One important advantage of this protocol over those based on RNPs is that fully RNA-based methods are usually more affordable when translating to the clinic,^2^ and mRNA can be synthesized in-house with minimal equipment. This methodology has been optimized for the specific *TRAC* and *TRBC* sgRNA sequences described in the protocol. Alternative sgRNAs with different target sequences (either within *TRAC* and *TRBC* or within other loci) must be tested and/or optimized to achieve the greatest knockout efficiency possible. Moreover, it may be necessary to optimize the electroporation settings when using different electroporation systems as the one described here.

## Troubleshooting

### Problem 1

Low purity or yield of CD8^+^ T cells after magnetic-activated cell sorting (related to step 17 before you begin).

### Potential solution

One of the possible causes of low yield of CD8^+^ T cells is an insufficient amount of beads added to the PBMC before magnetic-activated cell sorting. To avoid this, correctly calculate the volume of beads needed and resuspend or vortex the beads prior to adding to the cell suspension. When added to the cell suspension, mix well. A possible cause of low purity could be the obstruction of the LS column by bubbles. Avoid the formation of bubbles when transferring the labeled cells to the LS column. Lastly, the presence of RBC or dead cells can also affect the purity of the isolated CD8^+^ T cells. If the PBMC sample contains RBC, consider treating the PBMC sample with RBC lysis buffer. The presence of cell aggregates due to cellular debris can be avoided by adding DNase-I (50 UI/mL) to the PBMC sample.

### Problem 2

Low yield of mRNA after IVT (related to step 34).

### Potential solution

One of the main reasons of low mRNA yield after *in vitro* synthesis is degradation by RNase. Although a RNase inhibitor is present in the Enzyme Mix of the mMESSAGE mMACHINE T7 transcription kit, it can only inactivate trace RNase contaminations. Therefore, production of IVT mRNA demands a dedicated RNA bench cleaned to remove the RNases and RNase free pipettes. In addition, the DNA template used for IVT can be contaminated with residual RNase A from the miniprep or introduced RNase from restriction enzymes. The resulting mRNA appears degraded and forms a smear on the quality control gel electrophoresis. In this case, proteinase K treatment of the DNA template to remove residual RNase before IVT is recommended. Lastly, a low yield can be caused by the degradation of the template DNA itself. Correct handling and storage of the linearized plasmid DNA is important.

### Problem 3

Low viability and yield after thawing of CD8^+^ T cells (related to step 41).

### Potential solution

When thawing cryopreserved cells, a fast-thawing method is important because of the toxic effect of DMSO at room temperature on cells. Adding thawed cells to an excess of prewarmed CTL medium dilutes DMSO to a non-toxic concentration. By centrifugation at 480 × *g* for 5 min at 19°C–22°C and resuspension in new CTL medium, DMSO is washed away. Additionally, to avoid more loss of T cells ensure that cryopreserved cells are recovered from the cryopreservation. Therefore, wait at least 4 h prior to culturing T cells.

### Problem 4

Loss of CD8^+^ T cells after activation (related to step 47).

### Potential solution

Anti-CD3 mAbs combined with anti-CD28 mAbs, IL-2 and IL-15 are strong T-cell activators and stimulate the expansion of CD8^+^ T cells. However, activation-induced cell death can occur when activating T cells. Activation-induced cell death can be avoided by adjusting mAb and cytokine concentration, duration of the stimulus and seeding density of the T cells.^9^ When too many cells die due to activation-induced cell death, keep the cells in culture until sufficient numbers of cells are reached.

### Problem 5

Low TCR KO efficiency (related to step 82).

### Potential solution

Low TCR KO efficiency can be caused by a poor quality of eSpCas9(1.1)-P2A-EGFP mRNA or the sgRNAs. When electroporating cells with RNA, it is important to keep the RNA on ice and work fast. When the RNA is added to the electroporation cuvette, proceed directly with the electroporation to avoid RNA degradation. Additionally, aliquoting RNAs is important to avoid repeated thaw-freezing cycles of the RNAs which can damage and destabilize the RNA. In general, instrument defects could be the cause of poor electroporation efficiencies. In this context, make sure to regularly perform quality controls of the instrument.

## Resource availability

### Lead contact

Further information and requests for resources and reagents should be directed to and will be fulfilled by the lead contact, Eva Lion (eva.lion@uantwerpen.be).

### Materials availability

There are restrictions to the availability of pST1 plasmids generated due to a material transfer agreement for the pST1 plasmid backbone. All information about materials can be addressed to and will be addressed by the lead contact.

## Data Availability

This study did not generate datasets or code.
